# Fungi as source for new bio-based materials: a patent review

**DOI:** 10.1186/s40694-019-0080-y

**Published:** 2019-10-26

**Authors:** Kustrim Cerimi, Kerem Can Akkaya, Carsten Pohl, Bertram Schmidt, Peter Neubauer

**Affiliations:** 1 Chair of Bioprocess Engineering, Institute of Biotechnology, Faculty III Process Sciences, Technische Universität Berlin, Ackerstrsse 76, ACK24, 13355 Berlin, Germany; 2 Chair of Applied and Molecular Microbiology, Institute of Biotechnology, Faculty III Process Sciences, Technische Universität Berlin, Gustav-Meyer-Allee 25, 13355 Berlin, Germany

**Keywords:** Filamentous fungi, Fungal composite material, Biomaterial, Fungal leather, Textile, Packaging material, Automotive industry

## Abstract

**Background:**

The circular economy closes loops in industrial manufacturing processes and minimizes waste. A bio-based economy aims to replace fossil-based resources and processes by sustainable alternatives which exploits renewable biomass for the generation of products used in our daily live. A current trend in fungal biotechnology—the production of fungal-based biomaterials—will contribute to both.

**Results:**

This study gives an overview of various trends and development applications in which fungal mycelium is used as new and sustainable biomaterial. A patent survey covering the last decade (2009–2018) yielded 47 patents and patent applications claiming fungal biomass or fungal composite materials for new applications in the packaging, textile, leather and automotive industries. Furthermore, fungal-based materials are envisaged for thermal insulation and as fire protection materials. Most patents and patent applications describe the use of different lignin- and cellulose-containing waste biomass as substrate for fungal cultivations, covering 27 different fungal species in total. Our search uncovered that most patent activities are on-going in the United States and in China.

**Conclusion:**

Current patent developments in the field suggest that fungal bio-based materials will considerable shape the future of material sciences and material applications. Fungal materials can be considered as an excellent renewable and degradable material alternative with a high innovation potential and have the potential to replace current petroleum-based materials.

## Introduction

Filamentous fungi are known as production organisms in biotechnology and have become indispensable in research and industry. Today, fungi are not only used for human consumption, but also fungal enzymes are widely used in the food, biofuels and detergent industries and fungal bioactive compounds are applied in veterinary and human medicine [1]. The metabolic products of fungal organisms are interesting for research and industry, and also the mycelial structure of filamentous fungi is moving into the focus of new areas of application. Given the experience to farm edible fungi on plant or animal waste material that is utilized by the fungus as the substrate [2], one extension that became attractive was the fabrication of structures that contain biomass material as a filler that is glued together by fungal mycelium [3]. In this regard, mycelia of filamentous fungi digest e.g. lignocellulosic materials and form entangled networks for mechanical strength and other properties [4], this opening new ways for the use of fungi. It is well known that petroleum-based polymers release carbon dioxide along the entire value chain. The use of mycelium-based products is regarded as biodegradable and sustainable and contributes to the transformation to sustainable economy, which is one of our challenges in society today [5]. In contrast to today’s fossil-based economy, which uses linear streams that lead to the depletion of fossil resources, circular economy approaches allow cycles to be closed by novel recyclable materials that can be generated from waste and secondary streams [6]. Sustainable product development and promising applications of fungi can be found in the textile industry, the packaging industry, as isolation material and in the automotive industry with different properties in terms of hydrophobicity, low or high density, insulation or non-flammability. The aim of this review is to collect and evaluate researched patent information that covers the years 2009–2018 to identify patents that consider the production and processing of fungal mycelium for novel applications. These patent searches serve to identify new technologies. Patents regarding the use of fungi in food production, as an already established industry, are not considered in the review.

## Results

### Patent search

**Fig. 1:**
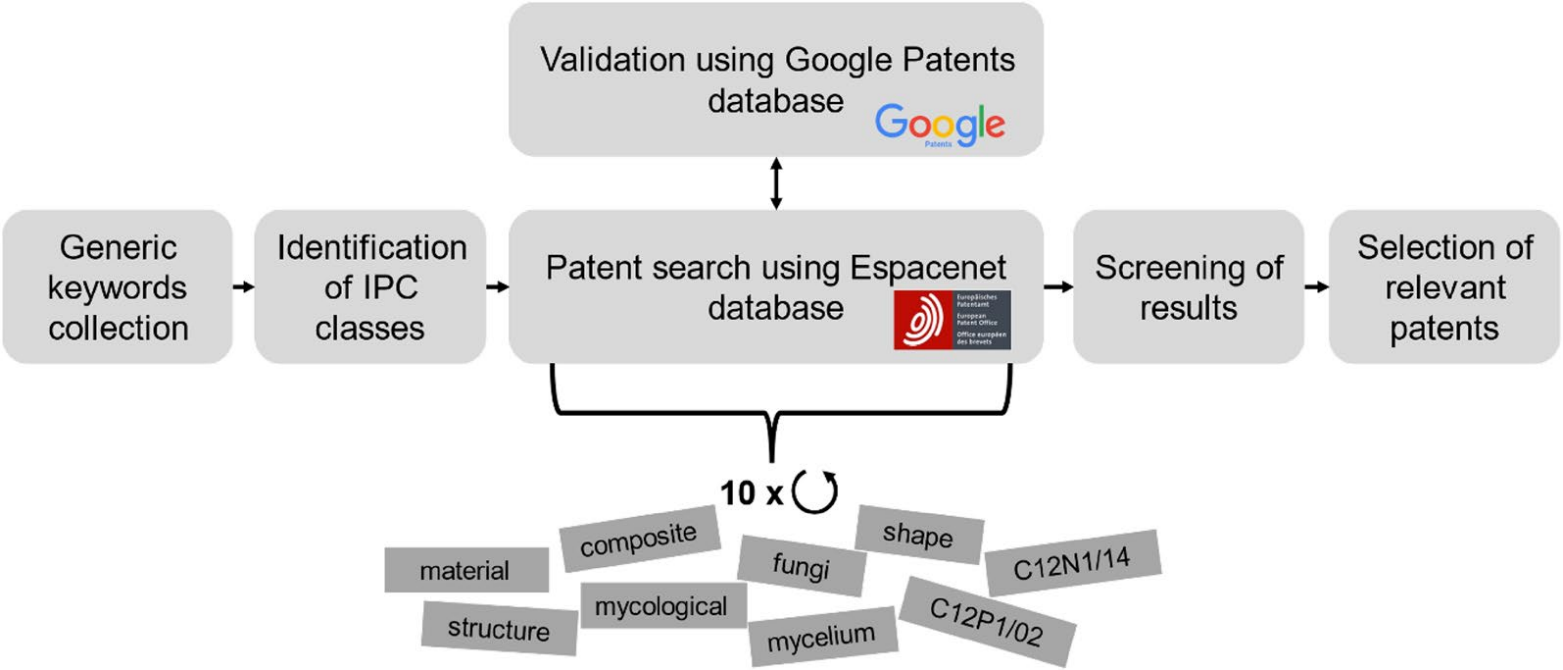
Open patent search and general workflow. Generic keywords and the relevant IPC classes were identified through screening of initially four different patents related to fungi as material usage. Patent search was carried out by using the European database Espacenet and additional validation by using Google Patents. A total of 10 search queries were carried out and the results were screened for relevant patents

Our study was based on a keyword search approach in different classes of the International Patent Classification (IPC) system^1^ (Fig. 1).

A total of 47 patents was found that cover the use of fungal materials in various areas. Patents applied for or granted in several countries were separated after the first filing or grant date, with only the first filing date being considered. In the following, we will give an overview of the patent distribution in terms of time and country and the areas of application described in the patents. Finally, we will summarize the substrates and organisms used.

### Analysis of patent situation

**Fig. 2:**
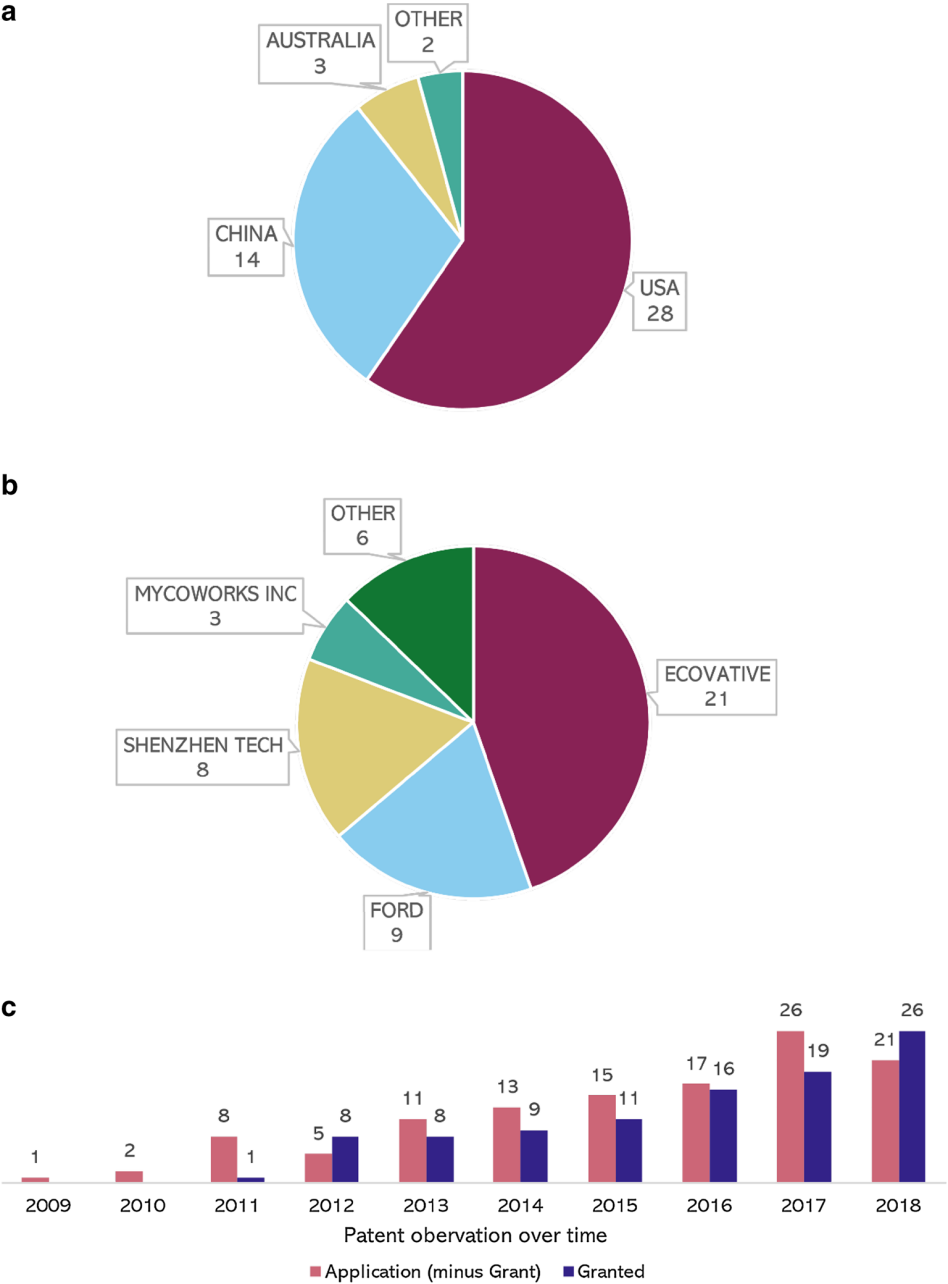
Distribution of the number of patents following different criteria. The information was extracted from the data of the patent search. **a** The figure shows the countries where the selected patents were granted. In case of multiple publications, the country of earliest granting is mentioned. The pie size for each country corresponds with the total number of patents first applied or granted there. Other = Canada, Italy, Japan with each one patent. **b** The figure reveals the distribution of patent ownership. The pie size for each patent assignee corresponds with their part in the selected 47 applied or granted patents, Other = see Additional file 1: Table S1. **c** The figure represents the cumulative development of the number of patent applications and number of granted patents over time. The height of the bars correlates with the cumulative number of applied or granted patents per each year. The bars for patents applied for include all new applications without patents already granted, since an approved patent is by definition no longer an application

The majority of patents was filed in the USA with 28 patents, followed by China with 14 patents, Australia with three patents and Canada and Japan with one patent each (Fig. 2a). Notably, the majority of patents are owned by companies and not by universities. The company Ecovative Design LLC (Ecovative) is leading with a share of 45% of all patents found, followed by Ford Global Tech (Ford) with 19%, Shenzhen Zeqingyuan Tech Dev Service Co Ltd (Shenzhen Tech) with 17% and MycoWorks Inc. (MycoWorks) with 6%. The remaining 13% are distributed among individual companies with one patent each (Fig. 2b).

The number of relevant patent applications and granted patents increased considerably from 2009 onwards up to 2018. Between 2010 and 2012, Ecovative and Ford increasingly filed applications. From 2015 on, Chinese companies began filing patent applications as well. In particular, compared to the total patent number in 2016, the number of applications or approvals increased by 50% in 2018 (Fig. 2c).

### Selected applications of fungal-based materials

#### Packaging

With globalization of the world industry, the market of the packaging industry raised during recent years. The current standard industrial packaging materials, extruded polystyrene and expanded polyethylene foams, are mainly based on petroleum products, which have various disadvantages regarding high energy consumption during production, difficulty regarding degradation and therewith environmental pollution. Green bio-composites derived from 100% bio-based materials could offer a sustainable alternative to petroleum-based plastic packaging in a wide range of applications [7].

Especially using agricultural raw materials, such as corn stalks or wheat straw as substrates to be utilized by selected fungi strains, the Chinese company Shenzhen Teq Dev. developed packaging material based on fungal mycelium (Table 1, Pos. 3). The product has several beneficial effects for being biodegradable and low in weight. It also contributes to the recycling of agricultural products and by-products and the replacement of existing packing material, thereby reducing environmental pollution. The biomaterial also shows good elasticity and buffering performance, making it particularly suitable as a packaging material. The production of orange-red colored packaging material without the use of added pigments by using a strain of *Pycnoporus cinnabarinus* was also patented (Table 2, Pos. 23). This bright pigmented and environmentally friendly mushroom material could be used directly for the production of sea buoys, as it also has a high buoyancy.

**Table 1:** Patent applications filed between 2009 and 2018 on usage of fungal material but not granted yet

Index	Current assignee	Country	Patent number	Details
1	Dongguan Hopeway Packaging Tech Co Ltd (2018)	CN	CN108249037	Production method for organic packaging material
2	Shenzhen Tech (2017)	CN	CN106675069 (WO2018120823)	Production method for fireproof composite material based on rice straw as main substrate
3	Shenzhen Tech (2017)	CN	CN106633991 (WO2018120826)	Production method for fireproof composite material based on rice straw as main substrate
4	Shenzhen Tech (2017)	CN	CN106675070 (WO2018120825)	Production method for fireproof composite material based on rice straw as main substrate
5	Zhongshan Torch Polytechnic (2017)	CN	CN106635825	Production method for organic packaging material
6	Shenzhen Huanan Tech Transfer Centre Ltd (2017)	CN	CN106752013	Production method for organic packaging material based on corncobs as main substrate
7	Shenzhen Tech (2017)	CN	CN106633990	Production method for organic packaging material based on maize straw as main substrate
8	Shenzhen Tech (2017)	CN	CN106633992	Production method for organic packaging material based on corncobs as main substrate
9	Shenzhen Tech (2017)	CN	CN106633989	Production method for organic packaging material based on bagasse as main substrate
10	Univ Jiangxi Sci & Technology (2016)	CN	CN106148199	Production method for organic packaging material
11	Dongying Eglin Biotechnology Co Ltd (2016)	CN	CN105660176	Production method for dehydrated mycelium elements
12	Ecovative (2013)	US	US20130224840	Production method for fabricating mycelium panels for construction
13	Mycoworks, Inc (2018)	US	US20180148682	Production method for dehydrated mycelium elements using molding systems
14	Ecovative (2017)	AU	AU2015271912	Production method using dehydrated mycelium to fabricate stiff engineered composite
15	Ecovative (2017)	US	US20170028600	Production method using dehydrated mycelium to fabricate stiff engineered composite
16	Ecovative (2016)	US	US20160302365 (WO2016168563)	Production method for dehydrated mycelium in a roll-to-roll format
17	Ecovative (2017)	AU	AU2015271910	Production method using dehydrated mycelium to fabricate stiff engineered composite
18	Ford (2013)	US	US20130202855	Production method for dehydrated mycelium elements for outfitting vehicle interiors
19	Ecovative (2014)	US	US20140056653 (WO2014031810)	Production method and machine for filling 3D cavities with bulk fungal material
20	Ecovative (2016)	US	US20160302364	Production method for dehydrated mycelium elements using *Xylaria* species
21	Ecovative (2015)	US	US20150033620	Production method for dehydrated mycelium elements

The patent number refers to earliest granting of the invention, linked to current assignee and the respective country, where the patent was applied in. In case of multiple publications, the country of earliest applying is mentioned. If identical versions of the same patent were issued in several countries, the patent numbers for the given countries are given in parentheses without reference of the publication year

*US* USA, *CN* China, *AU* Australia, *Ecovative* Ecovative Design LLC, *Ford* Ford Global Tech, *Shenzhen Tech* Shenzhen Zequngyuan Tech Dev Service Co Ltd., *Mycoworks* Mycowork, Inc.

**Table 2:** Granted patents published between 2009 and 2018 on usage of fungal material

Index	Current assignee	Country	Patent number	Details
1	Ecovative (2014)	JP	JP5457194 (US9485917, ES2574152, CN101627127 AU2007333545, DK2094856, EP2094856, CA2672312)	Production method for a self-supporting composite material for organic packaging and firewall material
2	Beijing Zhongke Aobei Supersonic Wave Tech Res Inst (2018)	CN	CN106758447	Production method for ultrasonically treated textile fibers
3	Shenzhen Tech (2017)	CN	CN105292758	Production method for organic packaging material
4	Ford (2016)	CN	CN102329512	Production method for vehicle parts consisting of mycelium used as a mat
5	Ecovative (2017)	US	US9803171	Production method for dehydrated mycelium elements
6	Ecovative (2018)	US	US10144149	Production method for stiff mycelium bound parts for furniture and fixations
7	Rensselaer Polytech Inst; Ecovative Design LLC (2015)	AU	AU2013251269	Production method for a self-supporting composite material, which could be used for sound attenuation
8	Ford (2012)	US	US8227225	Production method for dehydrated mycelium elements for outfitting vehicle interiors
9	Mycoworks (2016)	US	US9410116	Production method for dehydrated mycelium elements
10	Mycoworks (2018)	US	US9951307	Production method for dehydrated mycelium elements for building or construction
11	Ecovative (2018)	US	US9914906	Production method for dehydrated mycelium elements
12	Ford; Automotive Components Holdings LLC (2012)	US	US8227233	Production method for dehydrated mycelium elements for outfitting vehicle interiors
13	Ford; Automotive Components Holdings LLC (2012)	US	US8227224	Production method for dehydrated mycelium elements for outfitting vehicle interiors
14	Ford; Automotive Components Holdings LLC (2012)	US	US8313939 (CN105365145, CN102329513)	Production method for dehydrated mycelium elements for outfitting vehicle interiors using injection molding
15	Ford; Automotive Components Holdings LLC (2012)	US	US8283153	Production method for dehydrated mycelium elements for outfitting vehicle interiors
16	Ford; Automotive Components Holdings LLC (2012)	US	US8298810	Production method for dehydrated mycelium elements for outfitting vehicle interiors using injection molding
17	Ford; Automotive Components Holdings LLC (2012)	US	US8298809	Production method for dehydrated mycelium elements to make hardened elongate structure as a load supporting member in vehicles
18	Ecovative (2016)	US	US9253889	Production method for growing electrically conductive tissue to form electric circuits composed of fungal mycelium
19	Ecovative (2016)	US	US9469838	Production method for dehydrated mycelium elements with biofilm treated mycelium to obtain bio-resin like strengthened compounds
20	Ecovative (2016)	US	US9394512	Production method for dehydrated mycelium elements
21	Ecovative (2018)	US	US9879219	Production method for dehydrated mycelium elements
22	Ecovative (2017)	US	US9714180	Production method for an absorbing and remediating composite material for contaminants
23	Ecovative (2015)	US	US9085763	Production method for dehydrated mycelium elements to form tissue morphology using *Pycnoporus cinnabarinus*
24	Ecovative (2011)	US	US8001719	Production method for dehydrated mycelium elements
25	Ecovative (2018)	US	US10154627	Production method for dehydrated mycelium elements
26	Ecovative (2018)	CA	CA2834095 (DK2702137, EP2702137, AU2012249802, JP5922225)	Production method for dehydrated mycelium elements

The patent number refers to earliest granting of the invention, linked to current assignee and the respective country, where the patent was granted. If identical versions of the same patent were issued in several countries, the patent numbers for the given countries are given in parentheses without reference of the publication year

*US* USA, *CN* China, *AU* Australia, *CA* Canada, *JP* Japan, *Ecovative* Ecovative Design LLC, *Ford* Ford Global Tech, *Shenzhen* *Tech* Shenzhen Zequngyuan Tech Dev Service Co Ltd., *Mycoworks* Mycoworks, Inc.

#### Automotive industry

Several patents describe the application of fabricated composite material based on fungal mycelium to be used to substitute petroleum-based products in the automobile industry. An example is a patent of Ford, which describes a specialized method for an injection moulding process (Table 2, Pos. 12). A mushroom-liquid mixture is injected into a mould, which is heated after filling. The finished casting can be used for vehicle interiors or creating tubular structures used as filling material as also mentioned by another patent of Ford (Table 2, Pos. 14). The fungal material could provide parts suitable for use in vehicles both structurally and aesthetically, eliminating the need for adhesive and reducing costs by eliminating manufacturing steps. This could reduce the use of plastics in vehicles. In addition, the finished injection-moulded parts are also biodegradable and could be produced from agricultural waste products.

#### Electrical circuit boards

Mushroom material can also be modified to have a wiring pattern for an electrical circuit on it. According to a patent of Ecovative, a sheet of mycelium is prepared by inoculating a substrate, mainly potato dextrose agar or broth, with a fungus in a solution containing metal salts of CuSO_4_, CuCI_2_ or AI_2_O_2_ (Table 2, Pos. 18). During the process, the metal salts are sequestered by thin mycelium sheets grown into a shape in correspondence to the wiring pattern.

#### Textile industry

Most patents regarding the potential use of fungal material for the textile industry refer to an invention by Dschida, which describes the usage of fungal pulp in the production of textiles [8]. The patent focuses on the specific use of fungal cell wall components as a raw resource for the production of textiles. Possible applications go beyond the production of textiles, and include utilization of the material for paper production, food wrapping, construction material e.g. fibreboard, absorbent materials, and even medical applications such as anti-microbial wound dressings and adhesive coatings. Due to its broad market potential, this patent provides a wide variety of applications. However, since the patent expired after 20 years at the end of 2018, it is expected that several new patents will emerge in the future to further develop this production method and utilize fungal material for additional textile related applications.

#### Other material-based applications

In the following various granted patents are described, whose applications are formulated more generally in their respective claims, but could nevertheless provide promising applications in the future. The production of a composite material using a selected fungal saprophyte strain capable of absorbing impurities by producing enzymes used for degradation of animal waste is described by an Ecovative patent granted in 2017 (Table 2, Pos. 22). For this purpose the mycelium is grown as a thin mat or in pellets in discrete particles. A post-treatment is applied to prevent moisture absorption into the panel. The fungus produces an enzyme that degrades specific chemical compounds including polycyclic aromatic hydrocarbons, thereby having the potential to bioremediate contaminants such as engine oils, fuels or pesticides. The spent substrate can easily be composted or disposed of. This approach provides several applications in animal bedding, in order to absorb liquid excrement, or in cars or machinery to clean spills of oils or lubricants, thus helping to prevent these potentially harmful substances from entering the environment.

The general production method of fungal composite material was described and patented in 2015 but provides alternative applications regarding the use of fungal material for producing organic insulation material with increased fire resistance to be applied in home construction as a firewall panel (Table 2, Pos. 7). The patent builds further on possible applications and proposed the usage of a panel, composed of a mycelia bonded core and stiff exterior faces, for doors, cubicle walls, and to replace conventional insulation material in house construction. One alternative described, but not claimed in 2014, was the use of panels consisting of fungal material in houses or automobiles for acoustical dampening (Table 2, Pos. 1). This could be a degradable alternative to polyurethane foams, which are often petroleum-based.

### Manufacturing methodology of fungal composite material

The general methodology of the production process is similar in all patents and focuses on the use of a selected fungal pure culture, a nutrient substrate that can be digested by the fungus, and in some approaches of a discrete material that does not serve as a substrate but adds stability to the final product. This mixture of growth medium, discrete material and inoculum is placed into a predetermined shape, in which the fungus grows into the final product in a controlled environment, taking on the shape of the cavity. During the growth process, the fungus develops hyphae, which then form a network of interconnected mycelium through and around the discrete material, thereby binding it together to form a self-supporting mycelium composite. Depending on the application, once fully ingrown in the mould, the product is either completely dried to prevent further growth, or partially dried to allow rehydration for growth of the fungus into adjacent parts, to bond them together to form one fabricated section as described in a patent by Ecovative (Table 2, Pos. 26).

In some applications, fungi are cultivated in flat shapes to form mycelium sheets, which then can be processed via cutting to obtain two-dimensional features or to form three-dimensional features by stacking and growing individual sheets together as mentioned in an invention in 2018 (Table 2, Pos. 21). Some approaches use plastic shapes to develop their desired products. Alternatively, the fabrication of the mould from sustainable, degradable substances such as bamboo or plant fibres was described, so that it can also be completely colonized by the fungus (Table 2, Pos. 25). This would contribute to the reduction of plastic waste.

### Overview of utilized substrates

The range of substrates used varies between those which have a defined composition with certain ingredients, and complex substrates whose origin, but not the chemical composition, are defined. In addition, there are substrates that are not degraded completely and are therefore necessary for features or function of the material. In principle, the requirements for the substrate are one or more carbohydrate sources and dissolved nitrogen and phosphorus. Most mentioned substrates therefore contain cellulose, lignin or both. In addition, sufficient water must be available. These substrates are particularly overgrown and metabolized by fungal organisms. Examples of used substrates are wheat straw, wheat bran, maize straw, bagasse, wood and wood-containing substrates such as sawdust or wood chips. In addition, substrates from wool, hemp or silk production can also be considered. Basically, all those organic and non-toxic materials that remain as agricultural and industrial waste from other production branches can be applied as feedstock [9]. As these materials still contain large amounts of cellulose, they are predestined for the cellulolytic metabolism of fungal organisms. Agricultural residues that accumulate as waste are one of the renewable and cellulose-rich biomass resources that are available in huge amounts [10].

An example of specific substrates directly related to the function of the finished component is described in a patent by Ecovative. This patent covers the production of an electrical circuit board which consists of fungal, fully grown material on the one hand, and on the other hand the metallic substances responsible for electrical conduction. These metals are already present in the substrate and contribute to the function of the finished component (Table 2, Pos. 18). Another example of an undefined substrate is described in a patent by Ford, using fibrous lignin-containing material coming from coconuts, maize, rice or silk (Table 2, Pos. 8).

Overall, it can be said that there is a wide variety of substrates to be used for the growth of fungal organisms. In particular fungal organisms are often able to utilize several substrate types and carbon sources. The abundance of the available substrates can differ regionally and it has been also shown that the properties of the mycelium-material are related to the substrates which were digested by the fungi [3].

### Overview of fungal species mentioned in patents

The use of the species mentioned in the 47 selected patents was analyzed based on the claims and the description of the patent. Some of the organisms mentioned can also be found in the overall description of a patent. They either appear in the claims or are only described as examples without further details in the claims. A total of 27 different organisms could be identified in the claims of granted and non-granted patents.

A total of 20 organisms have been found in granted patents (Table 3). Five organisms appear only in the claims of applications, so that these or their use have not yet been granted. Two organisms cannot be found in the claims of the particular application or grant, but were mentioned as examples in the overall description of the patent. The majority of the species mentioned in Table 3 belong to the systematic phylum of basidiomycetes. It has been shown that the mycelium of different species of basidiomycetes, such as *Trametes versicolor* and *Pleurotus ostreatus* show high strength and stiffness in the final product [11]. Only five fungal species belonging to the genera *Morchella* and *Xylaria* are assigned to the phylum of Ascomycetes (Table 3). *Xylaria* species have appeared exclusively in the patents that have not been granted until now. Most frequently, the use of the species *Ganoderma lucidum* and *G. orogenese* (five patents) and *P. ostreatus* (four patents) was described. The ability to form honeycomb resembling structures with flexible structural components on the one hand and with a certain strength on the other hand is described in 2011 for the organisms of the genus *Polyporus, Fomes* and *Ganoderma* (Table 2, Pos. 24). This could provide a possible explanation for the increased occurrence in the claims of the granted patents.

**Table 3:** Overview of fungal species mentioned in the selected patents

Phylum	Order	Species	Granted	Not-granted
Basidiomycota	*Agaricales*	*Agrocybe aegerita*	3	–
		*Agrocybe brasiliensis*	1	–
		*Coprinus comatus*	3	–
		*Flammulina velutipes*	1	1
		*Hypholoma capnoides*	1	–
		*Hypholoma sublaterium*	1	–
		*Lentinula edodes*	3	–
		*Macrolepiota procera*	–	–
		*Pleurotus djamor*	2	–
		*Pleurotus eryngii*	2	–
		*Pleurotus ostreatus*	4	2
		*Pleurotus ostreatus var. columbines*	3	–
	*Polyporales*	*Fomes fomentarius*	2	1
		*Ganoderma tsugae*	2	–
		*Ganoderma lucidum*	5	1
		*Ganoderma orogenese*	5	1
		*Grifola frondesa*	2	–
		*Piptoporous betulina*	2	1
		*Polyporus mylittae*	–	–
		*Pycnoporus cinnabarinus*	1	–
		*Trametes versicolor*	2	1
	*Russulales*	*Hericium erinaceus*	–	1
Ascomycota	Pezizales	*Morchella angusticeps*	1	–
	Xylariales	*Xylaria polymorpha*	–	1
		*Xylaria hypoxylon*	–	1
		*Xylaria filiformis*	–	1
		*Xylaria longipes*	–	1

The fungal strains were extracted from the selected patents regarding the patent status being granted or not-granted

## Discussion

Our patent search uncovered a significant increase in the number of patents related to the emergence of fungal applications in the material sector. Various companies and institutions are increasingly patenting in this field, particularly in the United States and China. In the course of the patent search it became clear that various methods are used by the patent applicants to keep their application for competitive reasons in the early stage undiscovered. During the application process it is possible, for example, that patents are assigned to IPC classes which at first glance are not clearly associated with fungal material and its use. These do not necessarily have to correspond to the IPC classes of the granted form of the patent, as these can still be amended. Thus, patents that did not appear in Espacenet due to the restriction of the IPC classes appeared when searching via Google Patents. In particular, this circumstance is to be owed to the uncomplicated search form of Google Patents, since it operates only with search terms. Notably, patent searches not necessarily represent the actual status of a development but that of at least 18 months ago due to current patent regulations. It is thus likely that till the end of 2018 a certain number of additional patents have been applied for, which are currently under examination and therefore not present in patent databases yet.

Since the patent search did not contain a specific application or applications regarding the use of fungi for food purposes or for pharmaceutical purposes, but rather patents which deal with the use of fungal mycelium, the analysis of the patent situation was not straight-forward. The use of the classical approach for patent searches would have led to an incomplete overview of the patent situation, which is often the case during patent surveys. Only the combination of different search words as done in the current study will lead to a comprehensive overview of the patent situation. Given that our survey has missed some patents and patent applications, the results presented here still clearly show a broad spectrum of newly identified applications for fungal material. Nevertheless, the application and market of fungal-based materials seems to be limited so far to a few stakeholders only.

## Conclusions

The analysis of the patent situation in the field of material-oriented applications of fungi convincingly shows that this area is currently a significant growth sector. Starting with applications in the field of art, the first concepts for the industrial production and use of mushroom-based materials in various industries are now emerging. The future will show to what extent and in which areas fungus-based materials can replace current materials through their sustainable production and re-use, as well as with new interesting properties. However, it is very clear that this field represents a great potential and necessity for applied transdisciplinary research.

## Methods

### Patent search

The search term syntax contained a part that refers to the fungus as organism, a part that broadly considers the use of the fungal material or its structure and an additional search term in this topic context. The IPC classes C12P1/02 and C12N1/14 were predominantly used, as they describe the production of components or compositions by fermentation with fungi. In addition, only those patents were selected that were classified either to the C12P1/02 or C12N1/14 classes. The time interval was limited to patents filed or granted in the period from 2009 to 2018. An overview of the search terms and the respective number of derived patents can be found in the supplements (Additional file 1: Table S1). The search query data were downloaded from Espacenet. The content of the search was re-examined and filtered to exclude patents related to food and pharmaceutical applications. The remaining patents were further examined regarding their subjects and actual claims. Finally, all relevant patents were compiled and re-analysed via Google Patents to analyse the chronological course of each patent, i.e. its first approval and countries where it was subsequently approved.

### Data extraction

The related patents for each search term were extracted and integrated into Excel data files. The exported information from Google Patents and Espacenet included: (I) Patent number, (II) Status, (III) Filing date, (IV) Application date, (V) First grant date, (VI) Later granted in, (VII) Inventor, (VIII) Assignee, (IX) Pending applications and (X) URL of Espacenet. This table served as the basis for further analysis and to develop a document-term matrix. This matrix was used for the patent analysis to filter the claims and descriptions of the patents according to different application terms. If both the patent applied for and the patent granted appeared in the search results at the same time, the granted patents were preferred, which means that each patent to be evaluated is unique in the list according to its current status. In the data extraction the different subcategories of the granted or filed patents (A1, A2 or B1, B2, C etc.) were recorded but in the evaluation, only the general difference between application (A) and granted (B and further) was made. Data analysis and visualization was executed using Microsoft Excel.

## Supplementary information

**Additional file 1.** List of all patents considered in the review.

## Data Availability

All data generated or analysed during this study are included in this published manuscript and its additional information file.

## Abbreviation

IPC: International Patent Classification

## Footnotes

1 https://www.wipo.int/classifications/ipc/en/ITsupport/ipc_systems_links.html.
